# Applying Low-Frequency Vibration for the Experimental Investigation of Clutch Hub Forming

**DOI:** 10.3390/ma11060928

**Published:** 2018-05-30

**Authors:** De’an Meng, Chengcheng Zhu, Xuzhe Zhao, Shengdun Zhao

**Affiliations:** 1 School of Mechanical Engineering, Xi’an Jiaotong University, Xi’an 710049, China; sdzhao@mail.xjtu.edu; 2 School of Mechanical Engineering, Northwest Polytechnical University, Xi’an 710072, China; 3 Department of Mechanical Engineering, National University of Singapore, Singapore 119260, Singapore; 4 School of Engineering Technology, Purdue University, 401 N. Grant Street, West Lafayette, IN 47906, USA; zhao636@purdue.edu

**Keywords:** clutch hub, vibration-assisted forming, load reduction, lubrication, surface quality

## Abstract

A vibration-assisted plastic-forming method was proposed, and its influence on clutch hub forming process was investigated. The experiments were conducted on a vibration-assisted hydraulic extrusion press with adjustable frequency and amplitude. Vibration frequency and amplitude were considered in investigating the effect of vibration on forming load and surface quality. Results showed that applying vibration can effectively reduce forming force and improve surface quality. The drop in forming load was proportional to the vibration frequency and amplitude, and the load decreased by up to 25%. Such reduction in forming load raised with amplitude increase because the increase in amplitude would accelerate punch relative speed, which then weakened the adhesion between workpiece and dies. By increasing the vibration frequency, the punch movement was enhanced, and the number of attempts to drag the lubricant out of the pits was increased. In this manner, the lubrication condition was improved greatly. The 3D surface topography testing confirmed the assumption. Moreover, vibration frequency exerted a more significant effect on the forming load reduction than vibration amplitude.

## 1. Introduction

Clutch hub is an important torque transmission component, which is widely used in automatic transmission. However, its complex shape and highly precise dimensions make it one of the most complicated parts in automobile manufacturing. The conventional forming method for clutch hub forming mainly focuses on hobbing [1] and hot forging [2,3,4]. Hobbing usually cuts the workpiece, which is time consumption. Hot forging needs to heat the materials, which has bad performance in cost and precision. For the production of lightweight, high-strength, and low-cost parts in the automobile industry, sheet–bulk forming, which combines sheet and bulk forming, has been proposed for shortening the process chain and achieving high-performance products [5,6,7,8].

Many researchers have attempted to apply sheet–bulk forming in clutch metal forming by experimental or numerical methods. Ko et al. [9] introduced a roll die forming method to improve the dimensional accuracy in manufacturing a clutch hub. An optimal clearance between the punch and the roll was determined by the finite-element method and experiments. Zhuang et al. [10] proposed a method that combines deep drawing and extrusion process for the production of parts, such as the geared drum, on a servo press. In his study, several slide motions, such as speed motion, oscillation motion, and step motion, were considered, and results showed that step motion positively affected the surface quality and thickness reduction. Meanwhile, Mori et al. [11,12] presented a resistance heating method for clutch hub forming to improve the formability of ultra-high-strength steel. By resistance heating, the fracture was prevented, and the clutch hub was produced successfully. Sun et al. [13] investigated the clutch hub extrusion processes with various extrusion ratios and preformed shapes through the AFDEX software (Metal Forming Research Corporation, Jinju, Korea). Their simulation results revealed that stress state is the main determinant of local material flowing behavior and can be used for improving the degree of tooth filling. Wu et al. [14] also used the finite-element method to analyze flanging and coining operations during clutch1 hub forming. Several experimental and simulation works have been conducted for the control of local thickness and prevention of cracks at selected process conditions. Park et al. [15] utilized the commercial finite-element software DEFORM-2D (Scientific Forming Technologies Corporation, Columbus, IN, USA) to optimize preformed shapes and tooling geometries in order to prevent excessive thinning and crack formation. And experiments for clutch hub forming processes were also carried out for the verification of analytical models. Lee et al. [16] described a 3D finite-element method involving nine deep drawing processes and three ironing processes for forming a clutch drum. They found that the punch shape, punch angle, and thickness reduction ratio influences ironing dimensional accuracy and forming load.

From the above descriptions, we learned that gear tooth formation undergoes an ironing process, and severe plastic deformation occurs during this procedure. The clutch hub is a thin-walled component, and large forming loads easily lead to damage on the surface and in internal parts. Owing to the development of servo press and hydraulic press [17,18], superimposing vibration to forming dies or workpieces during plastic processes has become a reality. Many scholars have devoted considerable effort for the development of vibration-assisted forming techniques, which enables the generation of good-quality products at a small forming load. Maeno et al. [19] applied pulsation to the forging process of stainless steel by using a servo press. Given the vibration, the elastic recovery between the die and the plate causes the liquid lubricant to automatically be fed into the gap, thereby greatly improving lubrication. Kriechenbauer et al. [20] described a novel technology for deep drawing on a servo press by superimposing low-frequency vibration between 10 and 50 Hz at a cushion and a press ram. Products with increased drawing ratios and no wrinkle were obtained through this technology. Matsumoto et al. [21] proposed a method for enhancing the friction condition in deep hole forming by controlling punch movement in pulsed and stepwise modes. Through this method, deep holes with high accuracy were produced. In these works, the most common effects induced by vibration were load reduction and surface quality improvement because of volume and surface effects. Travieso-Rodríguez et al. [22] proposed a vibration-assisted ball-burnishing process by attaching a vibrating module to a classical burnishing tool. The surface quality and process efficiency have been significantly improved with the assistance of external vibration energy. In the meanwhile, Jerez-Mesa et al. [23] also designed a ultrasonic vibration-assisted ball-burnishing tool for surface treatment of material Ti-6Al-4V. The vibration-assisted ball-burnishing technique showed a good performance both in surface quality and hardness improvements compared with traditional burnishing. Moreover, vibration also can affect the material property greatly. Liu et al. [24,25] presented a new method to produce ultrafine copper materials by using the ultrasonic. By applying ultrasonic vibration during upsetting procedure, the initial grain size drops from 50 μm to 100 nm. The statements above show vibration both has a significant effect on the interior and surface of the material, which is named volume effect and surface effect, respectively. The volume effect is manifested by the vibration-induced enhancements in dislocation movements and decreased flow stress levels [26,27]. The surface effect is attributed to the effect of vibration on friction condition in the interface between a workpiece and dies [28,29]. The volume and surface effects greatly influence forming load reduction. However, not all vibration-assisted plastic forming processes exert volume and surface effects. The result depends on the vibration frequency and amplitude [30].

To reduce the forming load and improve the quality of the clutch hub, we proposed a novel method for the application of vibration during traditional sheet–bulk forming. We developed a specialized device for vibration-assisted extrusion to investigate the effects of vibration on the clutch hub. Then, the effects of vibration frequency and vibration amplitude on forming load were experimentally investigated. The clutch hub surface was also examined with a 3D microscope. Based on the results, we propose a basic explanation for load reduction due to vibration.

## 2. Materials and Methods

### 2.1. Material Properties

Low-carbon sheet steel DC04 (produced by baosteel, Shanghai, China) was used, which is widely used in automobile manufacturing because of its good performance in stamping and drawing. The DC04 sheet’s thickness was 1.5 mm, with an average grain size of about 20 μm. The chemical composition of the material is shown in Table 1.

### 2.2. Process of Vibration-Assisted Clutch Hub Forming

**Figure 1:**
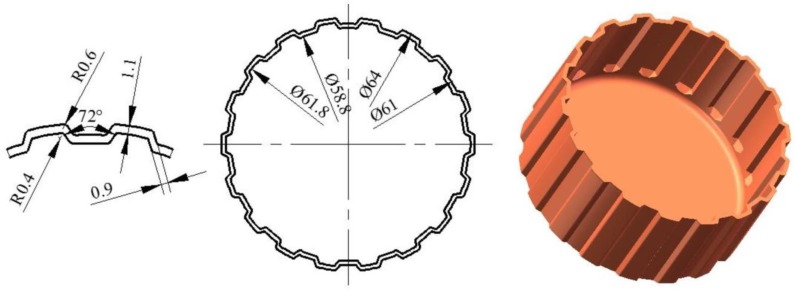
Clutch hub geometry dimensions (unit: mm).

The clutch hub studied in this paper was a periodic and symmetric cylindrical part with 18 teeth that are aligned circumferentially (Figure 1). The clutch hub is a key part and plays an important role in power transmission in automotive transmission systems. The outer and inner diameters of the drum are 64 and 58.8 mm, respectively. The tooth length was 22.5 mm. The top and side thicknesses of the tooth were 1.1 and 0.9 mm. Moreover, the maximal and minimal corner radii were 0.6 and 0.4 mm.

**Figure 2:**
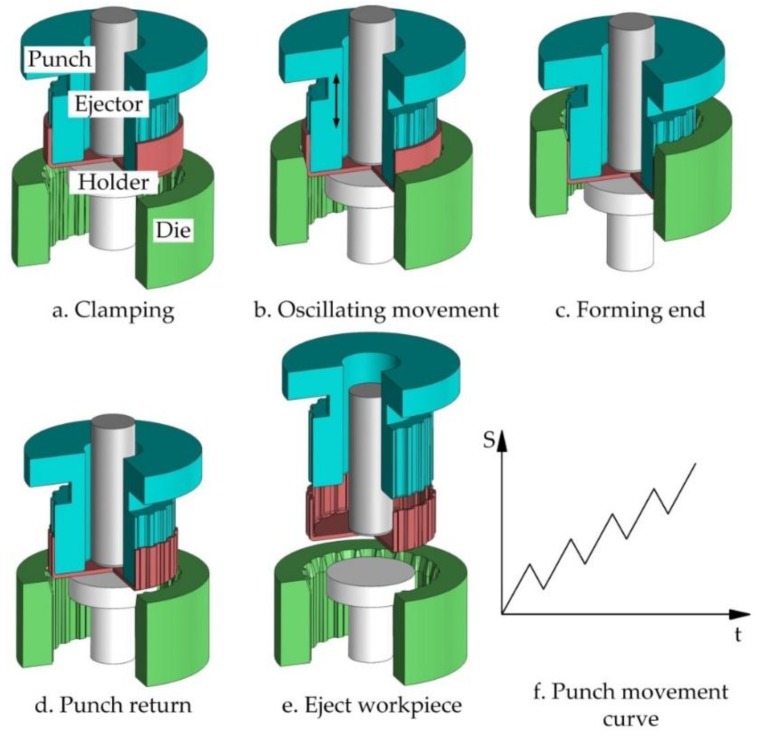
Procedure of clutch hub forming processing.

Figure 2 shows the vibration-assisted clutch hub forming process and includes five steps. The workpiece was a drawn cup with 1.3mm thickness and the sidewall was cut to a height of 20 mm. The first step was workpiece clamping, in which a workpiece was clamped by a holder and punched. Then, the punch carried the workpiece down the die inside in an oscillating manner. The punch was superimposed with low-frequency vibration during clutch hub forming. Its movement curve is shown in Figure 2f. This feed mode was named “forward-one-backward-half”. When the forming was ended, the punch reverted to its original state, and the workpiece returned with the punch because of friction. Lastly, the workpiece was ejected by the ejector. During the forming procedure, the die was superimposed at a reciprocating motion. In contrast to conventional cold forming processes, vibration-assisted forming decreases forming force and thereby improves product quality. Given the rebuilding of lubricating film during the reciprocating motion, the friction condition was improved.

### 2.3. Experiments

**Figure 3:**
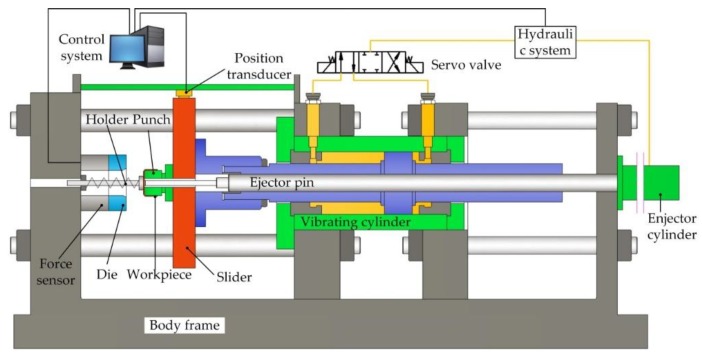
Schematic of a vibration-assisted hydraulic extrusion press.

Vibration-assisted clutch hub forming was conducted with the setup shown in Figure 3. The setup was composed of the control system, hydraulic system, vibration cylinder, ejector cylinder, servo value, displacement sensor, force sensor, and forming dies. The hydraulic system supplied a pressure range of 0–15 MPa, providing a maximum force of 300 kN. The control system can alter the vibration frequency and amplitude by changing the servo value’s switching frequency and interval. The servo value’s response time was less than 10 ms, and the maximum vibration frequency of the vibrating cylinder was 50 Hz. The mover of the displacement sensor was fixed with the slider, and its motion precision was ±10 μm. The force sensor was installed between the die and body frame for recording forming force in the axial direction. To investigate the influence of vibration frequency on the forming force, we set the vibration frequency to 30, 20, or 10 Hz. The average velocity of the punch was 5 mm/s. The lubricant used in the forming experiment was liquid lubricant, and the parameter was 0.485 PaS. To investigate the influence of amplitude on the forming force, we set the vibration amplitude to 0.2, 0.4, or 0.6 mm, where the vibration frequency was fixed at 20 Hz. After the forming experiments, the surface quality and 3D morphology of the formed parts were further analyzed by a color 3D laser microscope (VK9710K, Osaka, Japan).

## 3. Results

### 3.1. Microstructure

**Figure 4:**
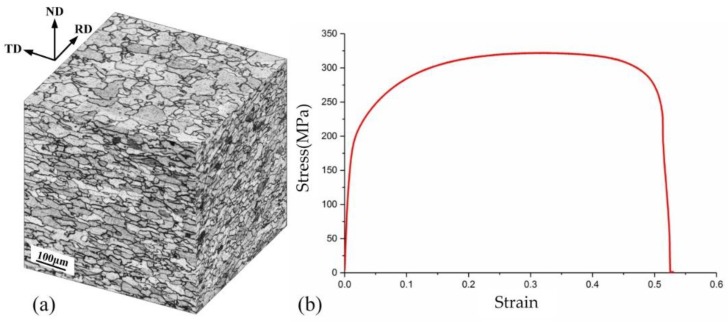
(**a**) The 3D microstructure and (**b**) stress and strain curve of the DC04 steel.

The material used in this study is a typical commercial sheet metal DC04. Figure 4a shows the 3D microstructure of the DC04 steel. Given the multiple rolling processes, the coarse grains were rolled and deformed into ellipsoids and showed long strip shapes, as viewed from transversal direction (TD) and rolling direction (RD). Many small grains were produced during the severe plastic deformation processing. The tension deformation behavior of DC04 is displayed in Figure 4b. The result shows that the average yield stress at 0.2% offset was 220 MPa, with an ultimate strength of 320 MPa and an elongation of 46.8%.

**Figure 5:**
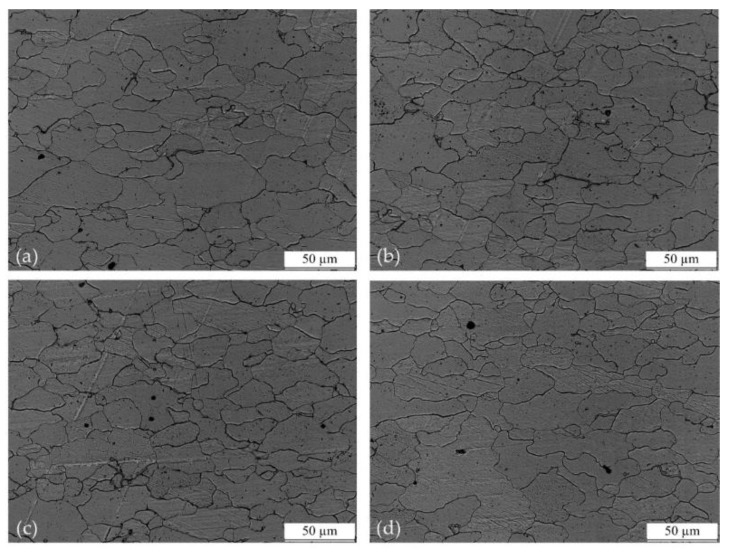
Cross-section microstructure applied different vibration frequencies: (**a**) Without vibration; (**b**) Applied vibration with frequency 10 Hz; (**c**) Applied vibration with frequency 20 Hz; (**d**) Applied vibration with frequency 30 Hz.

To investigate the vibration on the material microstructure, the formed parts were cut along the section. Four cases were taken into consideration: (a) without vibration; (b) applied vibration with frequency 10 Hz; (c) applied vibration with frequency 20 Hz and (d) applied vibration with frequency 30 Hz. The sections were polished with sand paper to mirror surface and etched with 4% nitric acid alcohol solution to reveal their microstructure. Then, metallographical observation was conducted using a Nikon LV150 microscope (Nikon Corporation, Tokyo, Japan). The microscope image results were shown in Figure 5. Then, the images were analyzed by the software Image Pro Plus (V6.0, Media Cybernetics Inc., Rockville, USA) and the average grain size was obtained, as shown in Table 2. There is no significant change in grain size between the four cases.

### 3.2. Forming Force along with Punch Displacement

**Figure 6:**
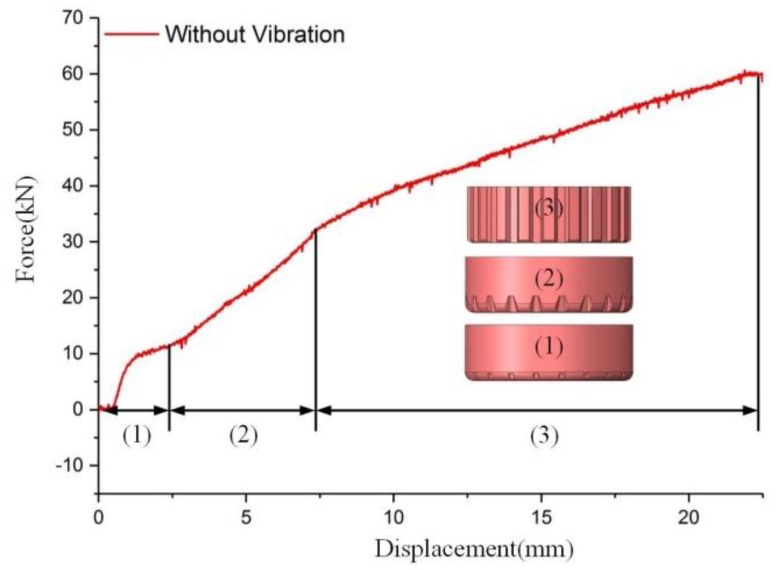
Load–displacement curve of clutch hub forming without vibration.

Figure 6 reveals the load–displacement curve without vibration. On the basis of the characteristics of the clutch hub, the load curve can be classified into three stages [10]: (1) elastic and microplastic deformation; (2) severe plastic deformation; and (3) gear tooth deformation. During stage (1), the workpiece contacted with the die and began to move inside the die. The material underwent elastic and microplastic deformation. The forming force increase was mainly caused by elastic deformation during this period. In stage (2), the material began to flow inside the die, and severe plastic deformation occurred. Plastic deformation was the main reason for the increased load. In stage (3), the gear tooth was gradually deformed. The contact area between the workpiece and die increased as the displacement increased. The friction force was proportional to the contact area; thus, the increase in forming force at this stage was mainly due to increased friction. The maximum load of clutch hub forming was 60.1 kN.

### 3.3. Influence of Vibration Frequency and Amplitude on Forming Force

**Figure 7:**
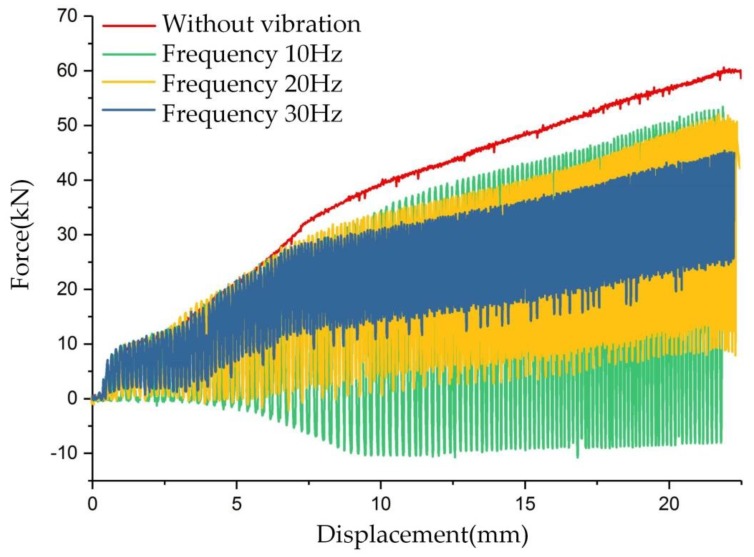
Comparison of load–displacement curves of clutch hub forming with different vibration frequencies.

The experiment results of vibration frequency on forming force are shown in Figure 7. Three kind values of frequency were considered, namely, 30, 20, and 10 Hz. The clutch hub forming process was separated into three stages, as described in Figure 6. In stages (1) and (2), we clearly observed that vibration causes fluctuations in the forming force, but the maximum forming force did not decrease. However, in stage (3), with the application of low-frequency vibration, an obvious load reduction occurred as the forming force fluctuated. The maximum load at the frequencies of 30, 20, and 10 Hz were 45, 51.4, and 55 kN, respectively. The corresponding load drop rates were 25%, 14.3%, and 8.3%. Because the servo valve used in this experiment was controlled in an open-loop method, the vibration amplitude was not well coupled with the frequency. When the vibration frequency increased, the commutation time of the servo valve was reduced and the amplitude subsequently decreased. On the basis of the displacement records obtained from the position transducer, the amplitude values were obtained, and the amplitudes at the frequencies of 30, 20, and 10 Hz were 0.16, 0.22, and 0.39 mm, respectively.

**Figure 8:**
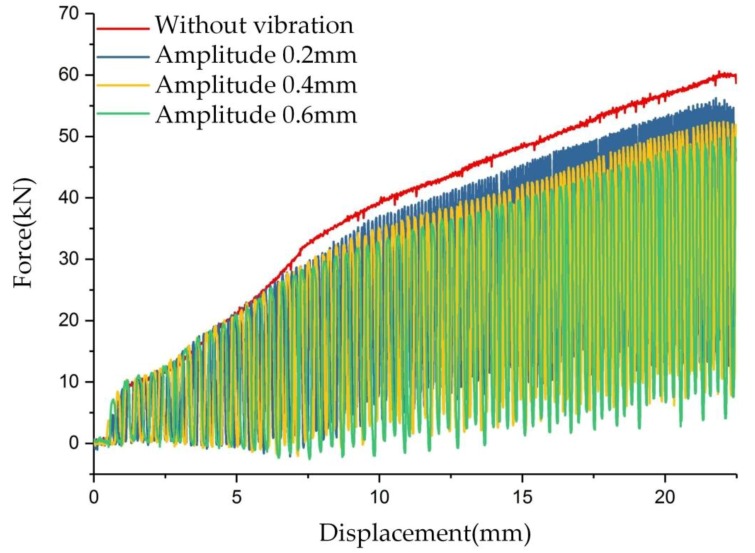
Comparison of load–displacement curves of clutch hub forming with different vibration amplitudes.

To investigate the influence of amplitude on the forming force separately, the vibration frequency was fixed at 20 Hz and three amplitudes, 0.2, 0.4, and 0.6 mm, were considered. The load–displacement curves of clutch hub forming with different vibration amplitudes are shown in Figure 8. In the forming stages (1) and (2), the forming force oscillated with the displacement increase and the upper limit of load did not show an obvious decrease. When the forming process moved into the stage (3), an obvious maximum load reduction was attained. The maximum load at the amplitudes of 0.2, 0.4, and 0.6 mm were 53.8, 51.8, and 49.8 kN, and the load drop rates were 10.3%, 13.6%, and 17%, accordingly. An increase in amplitude also positively caused the drop in forming load.

### 3.4. Influence of Vibration Frequency and Amplitude on Surface Quality

**Figure 9:**
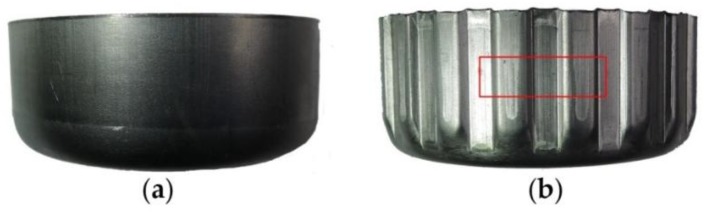
Surface appearance before and after the clutch forming process without vibration: (**a**) Before forming; (**b**) After forming.

Figure 9 shows the surface appearance of a clutch drum before and after the gear forming process. Before the gear forming process, the workpiece was a 1.3 mm-thick cylindrical part. After gear forming, the thickness of the workpiece wall was reduced to 1.1 mm. The gear tooth underwent severe plastic deformation, and obvious scratches appeared on the gear tooth surface, as shown by the red rectangle of Figure 9b.

**Figure 10:**
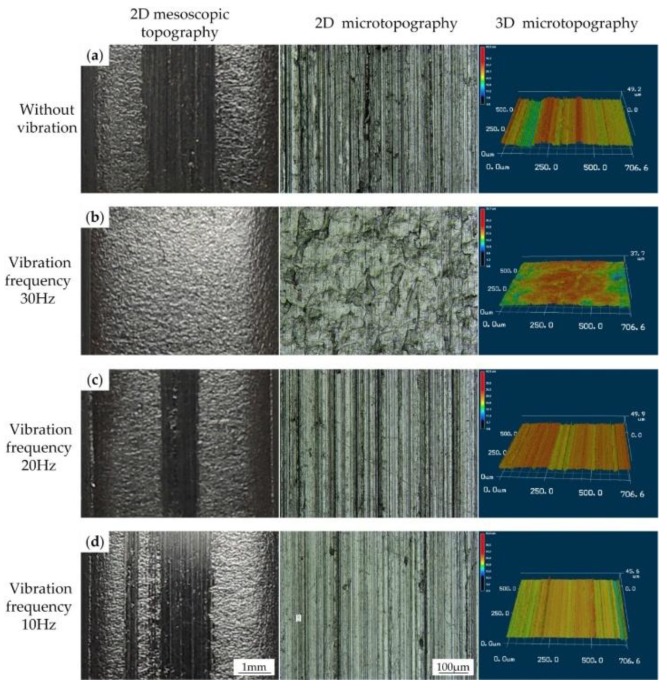
Comparison of the surface quality of clutch hubs formed under different vibration frequencies: (**a**) Without vibration; (**b**) With vibration at frequency 30 Hz; (**c**) With vibration at frequency 20 Hz; (**d**) With vibration at frequency 10 Hz.

**Figure 11:**
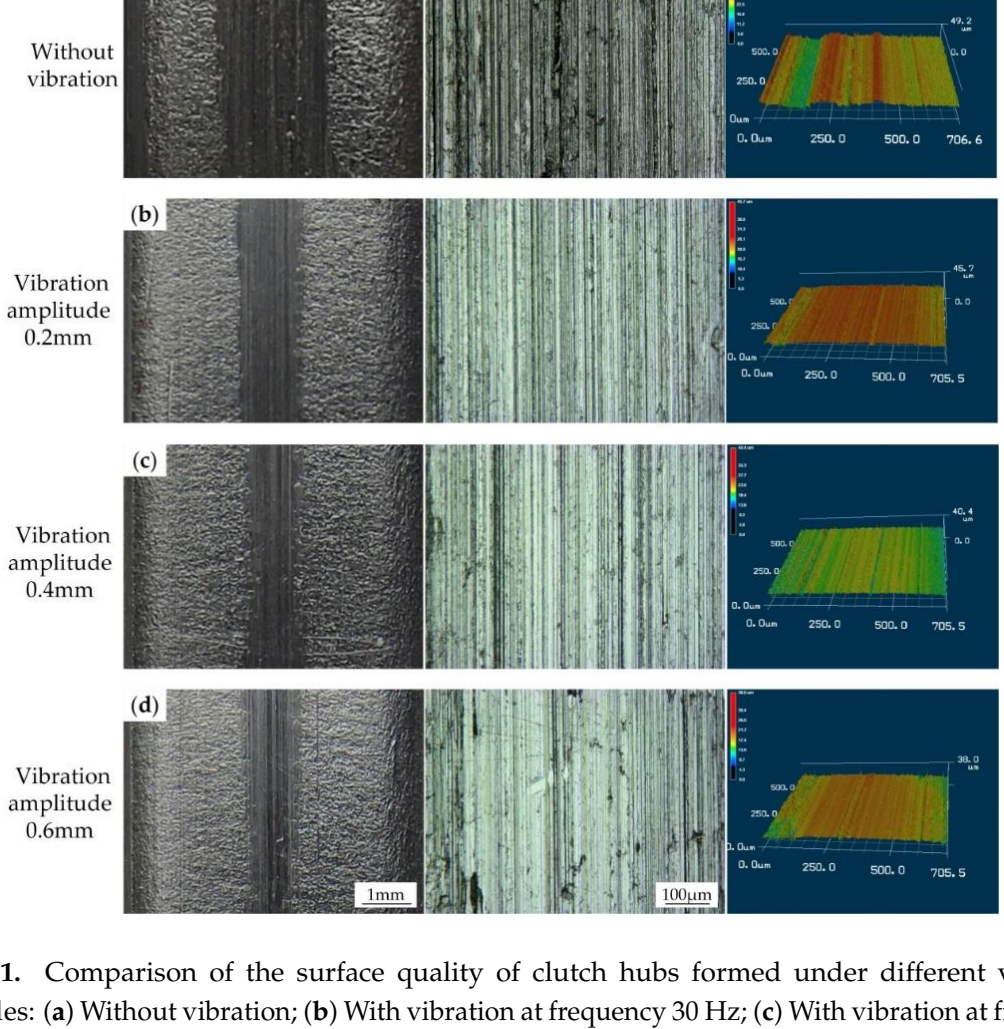
Comparison of the surface quality of clutch hubs formed under different vibration amplitudes: (**a**) Without vibration; (**b**) With vibration at frequency 30 Hz; (**c**) With vibration at frequency 20 Hz; (**d**) With vibration at frequency 10 Hz.

To investigate the influence of vibration on the surface quality, we conducted a series of microscopic experiments on a 3D laser microscope. The middle part of the gear tooth (red rectangle area showed in Figure 9b) was cut off from the formed part for the microscopic observation. The topography experiment results of the gear tooth surface are shown in Figure 10 and Figure 11. The 2D mesoscopic topography results showed obvious scratches appearing on the gear tooth surface in the absence of vibration. In Figure 10, when the vibration was applied on the punch, the scratch width on the gear tooth surface started to decrease. This result indicates that increase in vibration frequencies results in decrease in scratch width. When the frequency reached 30 Hz, the scratches tended to fade away. 2D and 3D microtopography results showed that scratches achieved a similar microstructure in the presence and absence of vibration and with vibration at the frequencies of 10 and 20 Hz. Numerous grooves were generated and revealed the occurrence of a severe plastic deformation on the gear tooth surface. However, under the vibration frequency of 30 Hz, the gear tooth surface was much flatter than in the other samples and did not retain a groove. 3D microtopography results showed that the asperities were flattened by the rigid tools, and many pits were retained on the surface. In Figure 11, the vibration frequency was fixed at 20 Hz. Increasing vibration amplitude form 0.2 mm to 0.6 mm has a positive effect on scratch width decrease. 2D and 3D microtopograpy results showed the surface roughness tends to become better as the amplitude increases.

## 4. Discussion

Obvious load reduction was observed during the vibration-assisted clutch hub forming in a wide range of frequency and amplitude. The reasons for load drop were classified into two types: volume and surface effects. In the volume effect, vibration is assumed to a kind of energy and can be absorbed by dislocations in the same manner as thermal energy. This effect reduces flow stress. In the surface effect, load reduction is attributed to the decrease in friction force caused by the relative motion in an interface.

As shown in Figure 6, clutch hub forming can be divided into three stages according to the curve’s local slope. In stages (1) and (2), the load fluctuated with the superposition of vibration, but the maximum load did not decrease. Given that the contact area between the workpiece and the die at the first two stages was small, the increase in forming force was mainly ascribed to elastic and plastic deformation. This notion means that the vibration only causes elastic unloading on materials, and vibration energy is not effectively transmitted to the interior of a material to cause flow stress reduction. In stage (3), the contact area between the workpiece and die gradually rises with the formation of the gear tooth. Frictional force is proportional to the contact area; thus, the increase in forming force at this stage was mainly due to the increase in friction. Figure 6 and Figure 7 reveal that the load reduction caused by vibration only occurred in stage (3). This result suggests that the phenomenon of forming force reduction in the vibration-assisted clutch forming process was mainly due to the change in friction condition and not the alteration of the material’s interior properties by the vibration. Additionally, the results in Figure 5 and Table 2 showed there was no significant change in grain size between the different vibration conditions, which confirmed our assumption that volume effect did not occur during vibration-assisted clutch hub forming process.

Figure 7 and Figure 8 show that the vibration frequency and amplitude positively affect the forming load reduction. However, in the experiment on vibration frequency on forming force, vibration amplitude is not a constant value and declines as the frequency increases. The load reduction phenomenon during this experiment suggests that vibration frequency exerts a more pronounced impact on load reduction than the vibration amplitude. Vibration frequency plays a more sensitive role in frictional force reduction during the clutch forming experiments.

**Figure 12:**
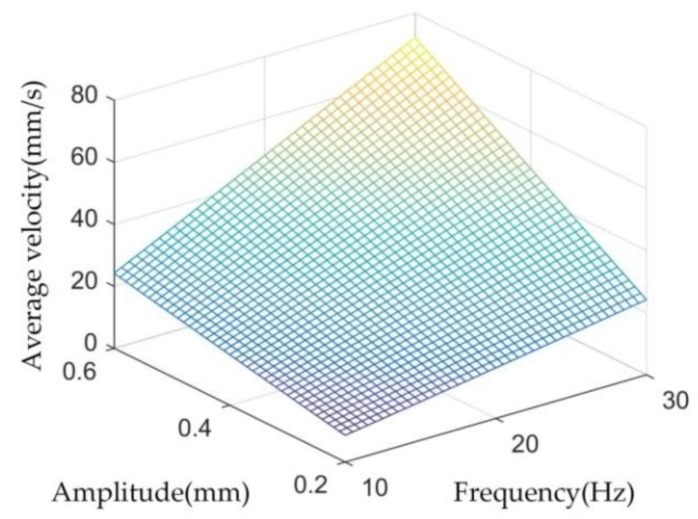
Influence of amplitude and frequency on the average velocity.

By analyzing the present results in detail, the reduction in forming force with the simultaneous application of vibration was found to be related with two factors, namely, the relative speed between the workpiece and die (related with frequency and amplitude) and the reciprocation times of punch (related with frequency). Given the theoretical and experimental results reported in prior studies [31,32,33,34], the relative speed significantly influences the frictional shear stress. Most of these works were based on the Stribeck curve [35]. In the friction regimes of boundary lubrication and mixed lubrication, high sliding speed results in decreased friction coefficient. The punch speed during vibration-assisted clutch hub forming can be decomposed into a constant and a cosine component and can be represented by the following equation:

(1) $$v=v_{0}+2\pi fAcos\left(2\pi ft\right),$$

where *v* is the punch speed, *v*_0_ is the velocity without vibration and its value is 5 mm/s, *A* is the vibration amplitude, and *f* is the vibration frequency. During one oscillation cycle, the punch speed’s applied vibration is larger than that without vibration in some specified intervals. The effective average speed can be used to evaluate the speed over a period of vibration and can be expressed as

(2) $$\bar{v}=\left(\int_{0}^{t_{1}}v+|\int_{t_{1}}^{t_{2}}v|+\int_{t_{2}}^{t_{3}}v\right)/T$$

where *T* is the vibration cycle, *t*_1_ and *t*_3_ are time intervals when the punch speed direction is positive, and *t*_2_ is time interval when the punch speed direction is negative. By combining Equations (1) and (2), the effective average speed $\bar{v}$ can be obtained, and its diagram is shown in Figure 12. The vibration amplitude and frequency both positively affect the increase rate of average speed. The Stribeck curve shows that the increase in average speed led to frictional stress reduction. This result thoroughly explains the findings in Figure 7.

**Figure 13:**
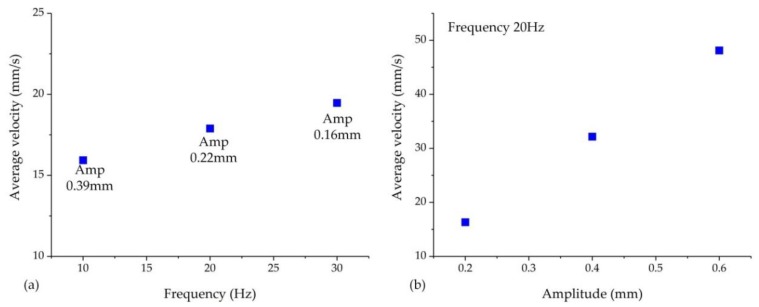
Influence of vibration frequency (**a**) and vibration amplitude (**b**) on the punch average velocity during vibration-assisted clutch hub forming.

**Figure 14:**
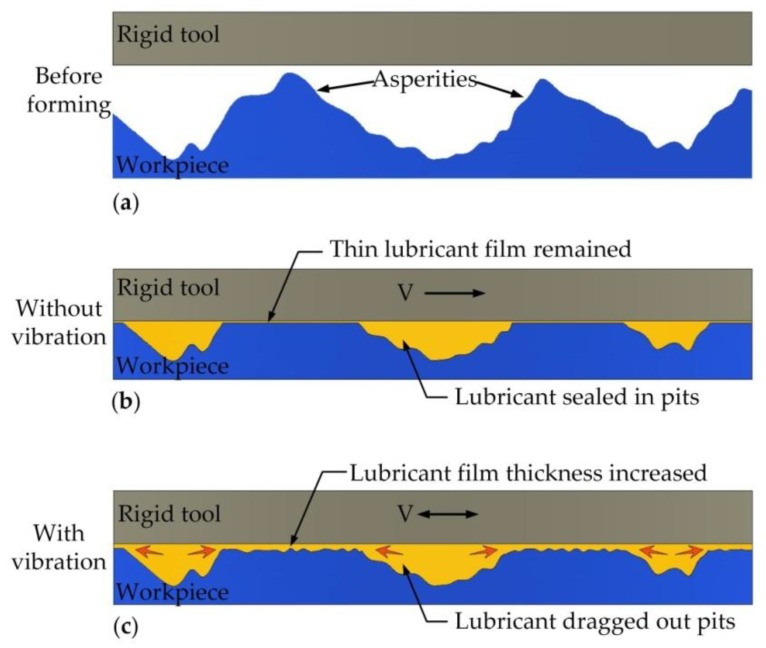
Comparison of lubrication during vibration-assisted forming with and without vibration: (**a**) Before forming; (**b**) Without vibration; (**c**) With vibration.

Similarly, in the corresponding experiments shown in Figure 7 and Figure 8, the average speed of punch is shown in Figure 13. Figure 13a displays that although the amplitude declined with increasing vibration frequency, the average velocity still increased. The amplitude exerted a more pronounced effect on the punch average velocity than that in the results in Figure 13a. However, the actual results shown in Figure 7 and Figure 8 were the reverse, and this observation implies that vibration frequency exerts an additional effect on frictional shear stress reduction rather than average velocity. The surface morphology results shown in Figure 10 reveal that increasing vibration frequency reduces the number of surface scratches, indicating that the reciprocation movement between the workpiece and die altered the lubrication condition. Thus, the higher the frequency is, the more obvious the effect is. This process is illustrated by Figure 14. Numerous asperities were randomly distributed throughout the workpiece surface. During plastic deformation without vibration, the rigid tool compressed the workpiece and flattened the asperities. Lubricant oil was sealed in the pits and an extremely thin lubricant film formed in the interface. During the vibration-assisted forming process, a rigid tool moved in an oscillation mode, and the relative velocity direction between the workpiece and die changed rapidly. The lubricant oil sealed in the microvalley was dragged out, and the lubricant film in the interface increased because of the vibration. The increase in vibration frequency raised the number of attempts for dragging out the lubricant oil from the pits. These notions explain why increase in frequency results in decreased load.

## 5. Conclusions

An improved vibration-assisted clutch forming method was proposed. The effects of vibration frequency and vibration amplitude on forming load reduction and surface quality were investigated by experimental methods. The main conclusions of this study are as follows:

(1) Increases in vibration frequency and amplitude positively affect forming load reduction.
(2) The effect of vibration frequency on the load drop was greater than that of the vibration amplitude.
(3) The forming load decreased with increasing vibration amplitude because the increase in vibration amplitude improved the relative speed between the workpiece and the blank and weakened the adhesion in the interface.
(4) The increase in vibration frequency raises the number of attempts for dragging out lubricant oil from the pits and in turn greatly widens the lubricant film thickness. The 3D microscope results confirmed this notion.

The range of vibration frequency and amplitude during experimental study were 10~30 Hz and 0.2~0.6 mm, respectively. The validity of the conclusions obtained in this paper is limited to the above conditions. This paper presented a basic explanation for the load reduction during vibration-assisted clutch forming. Nevertheless, a more rigorous and theoretical model must be developed in future research.

## Figures and Tables

**Table 1:** Chemical composition of DC04 steel.

C%	Mn%	Al%	P%	S%
0.07	0.3	0.02	0.02	0.025

**Table 2:** Average grain size with different vibration frequencies.

Without Vibration	Vibration Frequency 10 Hz	Vibration Frequency 10 Hz	Vibration Frequency 10 Hz
24.3 μm	25.2 μm	24.1 μm	25.6 μm
